# Trend in the detection rate of syphilis among older adults: time series analysis, Brazil, 2014–2023

**DOI:** 10.1590/S2237-96222026v35e20250900.en

**Published:** 2026-04-10

**Authors:** Gustavo Gabriel Oliveira, Anderson da Silveira Gonçalves, Heitor Wermann, Renan Garcia Artus, Felipe Groppelli, Nathiele Nunes da Silva Gonçalves

**Affiliations:** 1 Universidade Federal de Ciências da Saúde de Porto Alegre, Porto Alegre, RS, Brazil; 2 Atitus Educação, Porto Alegre, RS, Brazil

**Keywords:** Aged, Syphilis, Aging, Public Health Surveillance, Time Series Studies, Anciano, Sífilis, Envejecimiento, Vigilancia en Salud Pública, Estudios de Series Temporales

## Abstract

**Objective:**

To analyze the trend in syphilis detection among older adults in Brazil from 2014 to 2023.

**Methods:**

Analytical time-series study using data on acquired syphilis in individuals aged 60 years or older, obtained from the Notifiable Diseases Information System (Sistema de Informação de Agravos de Notificação, SINAN). Trend analysis was performed using segmented regression to calculate the Average Annual Percent Change (AAPC). Variables analyzed included sex, age group, and macroregion.

**Results:**

A total of 117,297 cases were reported (8.28% of total notifications). The mean detection rate was 59.80 cases per 100 thousand older adults, higher among men (77.39/100,000 versus 50.65/100,000 among women). The highest rates occurred in the 70–79-year age group (80.75/100,000), particularly in the South (114.88/100,000) and Central-West (102.68/100,000) regions. Until 2020, a significant national upward trend was observed (among women aged 70–79 years, AAPC 26.56; men aged ≥80 years, AAPC 23.71). Between 2020 and 2023, most groups showed stability, although increases persisted in subgroups, such as men aged 70–79 years in the Southeast (AAPC 20.63) and South (AAPC 16.14).

**Conclusion:**

Syphilis increased significantly until 2020 and subsequently stabilized at a high plateau. Gender and regional disparities were evident: mean rates were higher among men than among women and in the South and Central-West regions than at the national average. Persistent growth after 2020 highlights a public health problem requiring targeted sexual health policies.

## Resumen

**Objetivo:**

Analizar la tendencia de detección de la sífilis en personas mayores en Brasil entre 2014 y 2023.

**Métodos:**

Estudio analítico de series temporales con datos sobre sífilis adquirida en individuos de 60 años o más, obtenidos del Sistema de Información de Agravamientos de Notificación (Sistema de Informação de Agravos de Notificação, SINAN). El análisis de tendencia se realizó mediante regresión segmentada, calculando el promedio de cambio porcentual anual (Average Annual Percent Change, AAPC). Se analizaron variables de sexo, grupo etario y macrorregión.

**Resultados:**

Se notificaron 117.297 casos (8,28% del total de notificaciones). La tasa media de detección fue de 59,80 casos por 100.000 personas mayores, mayor en hombres (77,39/100.000 frente a 50,65/100.000 en mujeres). Las tasas más altas se observaron en el grupo de 70-79 años (80,75/100.000), en particular en las regiones Sur (114,88/100.000) y Centro-Oeste (102,68/100.000). Hasta 2020, se observó una tendencia nacional de crecimiento significativo (en mujeres de 70-79 años: AAPC 26,56; en hombres ≥ 80 años: AAPC 23,71). Entre 2020 y 2023, la mayoría de los grupos mostró estabilidad, aunque persistieron aumentos en subgrupos, como en los hombres de 70-79 años en el Sudeste (AAPC 20,63) y en el Sur (AAPC 16,14).

**Conclusión:**

La sífilis aumentó significativamente hasta 2020 y se estabilizó en niveles elevados. Las disparidades de género y regionales fueron evidentes: las tasas medias fueron mayores en los hombres que en las mujeres, y en las regiones Sur y Centro-Oeste, en comparación con la media nacional. El crecimiento persistente a partir de 2020 evidencia un problema de salud pública que requiere políticas dirigidas a la salud sexual.

**Ethical aspects**

This research used public domain anonymized databases.

## Introduction 

The aging of the Brazilian population has increased in recent decades, evidencing a demographic transformation in the country. The number of people aged 60 years or older has grown, reflecting increased life expectancy (1). This phenomenon poses challenges for adapting public health policies, necessitating changes to ensure quality of life for this segment of the population. The growth of the older adult population can be attributed to several factors, including declining mortality, advances in medicine, and improvements in primary health care. 

Data indicate that this trend of population growth is expected to continue in the coming years, placing pressure on health and social security systems, which will need to adapt to meet the new demands of a population with a range of chronic and acute diseases, disabilities, and needs for specialized care (2). The increase in the older adult population has been accompanied by growth in syphilis infections, as lack of knowledge about sexually transmitted infections (STI), combined with risk behaviors, predisposes older adults to infection (3,4). Despite this, few studies include this group in assessments of the topic (5). 

In Brazil, an increasing trend in the syphilis detection rate among older adults was identified between 2011 and 2019 (6). In 2020, the prevalence of syphilis was estimated, demonstrating a detection rate of 12.84 cases per 100 thousand Brazilians, with higher rates among men in the South and Southeast regions (7). However, studies have not yet compared data from before and after the COVID-19 pandemic to analyze the effects of this event on syphilis trends, which limits understanding of the national panorama and therefore justifies the present study. The pandemic may have altered sexual behaviors among older adults, as well as syphilis diagnosis and treatment. Therefore, evaluating periods with a focus on COVID-19 is essential to determine whether the previously observed increasing trends were maintained and whether regional or population-level changes occurred among older adults, the focus of this study. 

## Methods 

### Study design

This is an analytical time series study conducted to examine the trend in syphilis detection rates among older adults (aged 60 years or older) in Brazil from 2014 to 2023. The units of analysis were disaggregated at the subnational level (27 states, including the Federal District) and at the macroregional level (North, Northeast, Southeast, South, and Central-West), according to the criteria defined by the Brazilian Institute of Geography and Statistics (*Instituto Brasileiro de Geografia e Estatística*, IBGE). As all available records from the Notifiable Diseases Information System (*Sistema de Informação de Agravos de Notificação*, SINAN) were included, no sample size calculation was performed, as the analysis was conducted on the complete universe of notifications, representing a total population approach using all data available for the study period. This strategy maximizes the scope and statistical power of the analysis and provides a national overview of the condition based on official epidemiological surveillance data. The final dataset generated for this analysis, including the compiled cases and population projections, was shared in a public repository (8).

### Data source

The epidemiological data on acquired syphilis in individuals aged 60 years or older were obtained from SINAN, a system managed by the Ministry of Health (MS) and fed by municipal and state health services. Syphilis notification is mandatory and is performed by trained health professionals (nursing technicians, nurses, and physicians) responsible for patient diagnosis and care. Notification is carried out using the Individual Notification Form and the Investigation Form, both standardized by the Ministry of Health, in health care services at all levels of care. The information is entered into the computerized systems e-SUS/SinanNet and consolidated by municipal, state, district, and federal health authorities. The case definition followed the criteria established in the Health Surveillance Guide (*Guia de Vigilância em Saúde*, GVS), published by the Ministry of Health in 2023. Detection of treponemal antibodies (rapid or laboratory test) was considered, and confirmation with a nontreponemal test showing a significant titer.

### Study population

The study population comprises older adults (≥60 years) reported in SINAN between 1 January 2014 and 31 December 2023. Analyses were conducted by place of residence of the reported case, respecting Brazil’s state and macroregional divisions. All confirmed notifications of acquired syphilis in individuals aged 60 years or older were included. Notifications with negative or inconclusive diagnoses or with inconsistencies in the “age group” field, such as notification forms with this field left blank, were excluded.

### Variables 

The dependent variable was the detection rate of acquired syphilis among older adults. Independent variables included: sex (male and female); age group (in years, 60–64, 65–69, 70–79, and ≥80); race/skin color (White, Asian, Brown [Brazilian mixed race], Indigenous, and Black); and education level (illiterate, elementary, secondary, and higher education); region of notification (North, Northeast, Southeast, South and Central-West); and case outcome (cure or death from syphilis or other cause). Ignored or missing data were retained and classified as “ignored”, allowing assessment of data completeness and minimizing information loss.

### Population estimates and rate calculation

Population estimates for the denominator of detection rates were obtained from the 2010 and 2022 demographic censuses of the IBGE, using the values provided for each age group mentioned above, by sex and state. For intercensal years (2014–2021), exponential interpolation was applied, and for 2023, extrapolation was performed based on the 2022 census values, using the average annual geometric growth rate (r), calculated by sex and age group for each state according to the formula: r=((P2022/P2010)^(1/12)) - 1. The population projection for each state was estimated as: Pt=P0×(1+r)^t, where P0 is the population from the 2010 census and t is the number of elapsed years. This method was adopted to ensure consistency between the two census counts, as the IBGE intercensal estimates conducted in 2018 showed significant discrepancies compared with the 2022 census. These differences may be explained by changes in the agency’s migration forecasts, which were particularly affected by the COVID-19 pandemic between 2019 and 2022. There were also variations between regional life expectancy projections and actual population longevity. This approach was chosen to achieve greater stability and alignment with the observed demographic trend. Detection rates were expressed per 100 thousand older adults.

### Statistical and temporal trend analysis

The temporal trend of detection rates was assessed using segmented regression (Joinpoint Regression Program, version 5.4.0). The model identifies significant inflection points and estimates Annual Percent Change (APC) and Average Annual Percent Change (AAPC), with 95% confidence intervals (95%CI) and a p-value<0.05. Other comparative approaches, such as the Autoregressive Integrated Moving Average (ARIMA) or the General Algebraic Modeling System (GAMS), were not considered because segmented regression is superior for detecting APC and is the only method that statistically identifies trend changes, yielding a distinct, easily interpretable APC for each linear segment. 

An inflection point in 2020 was prespecified, with an epidemiological justification based on the impact of the COVID-19 pandemic on health surveillance and syphilis diagnosis. This choice enabled comparison of trends across two segments: 2014–2020 and 2020–2023. No additional inflection points were required in the analysis, given the linear growth observed in the remaining periods. The program’s grid search function with permutation testing was used to assess whether including the fixed point improved model fit, and it was confirmed. Assumptions of residual independence, homoscedasticity, and absence of temporal autocorrelation were evaluated. Sensitivity analyses were conducted to assess the potential influence of extreme values and the robustness of estimates over short intervals within the 2020–2023 period. The final trend classification used the following criteria: “increasing” if AAPC>0 and p-value<0.05; “decreasing” if AAPC<0 and p-value<0.05; and “stable” if p-value≥0.05. Data analysis and database management were performed using Microsoft Excel and the Joinpoint Regression Program, ensuring reproducibility and methodological transparency in accordance with the Strengthening the Reporting of Observational Studies in Epidemiology (STROBE) and Reporting of studies Conducted using Observational Routinely-collected Data (RECORD) guidelines. 

## Results 

From 2014 to 2023, a total of 117,297 cases of acquired syphilis were reported among older adults (60 years or older), corresponding to 8.28% of all notifications in Brazil (n=1,415,531).

The overall mean detection rate during the study period was 59.80 cases per 100 thousand inhabitants, higher among males (77.39/100,000). The mean age at notification was 68.38 years (±6.5). The 60–64-year age group was the most represented (38.4% of men; 38.23% of women), followed by the 65–69-year group (26.99% of men; 25.93% of women).

Regarding sociodemographic variables, a substantial proportion of data were missing. For race/skin color, 14.69% of notifications lacked this information; among the completed entries, 40.57% of individuals were reported as White and 33.23% as Brown. For education level, 41.20% of records were blank; among those completed, 14.36% indicated incomplete elementary education, and 2.42% higher education.

Clinical outcome was not reported in 50.46% of notifications. Among cases with this information, 48.98% resulted in a cure. Deaths directly attributed to syphilis represented 0.08% of cases, and those due to other causes accounted for 0.46%.

The South and Central-West regions showed detection rates above the national average (Table 1 and Supplementary Figure 1). In the South, rates were 71.83/100,000 inhabitants (60–64 years) and 68.08/100,000 (65–69 years), whereas the Brazilian average was 60.65 and 57.01, respectively. In the Central-West, rates were 60.25 (60–64 years) and 55.27 (65–69 years). Peak detection occurred in the 70–79-year age group, in which regional rates also exceeded the national average (80.75). Among individuals aged 80 years or older, values in the South (47.81) and Central-West (44.79) remained above the national mean (40.79).

**Table 1:** Average detection rate (per 100,000 older adults), by region and age group. Brazil, 2014–2023 (n=32,281,479)

Region	60–64 years	65–69 years	70–79 years	≥80 years
Brazil	60.65	57.01	80.75	40.79
Central-West	60.25	55.27	102.68	44.79
Northeast	44.81	42.91	74.45	28.15
North	69.57	62.39	106.75	43.18
Southeast	56.12	52.43	74.86	36.63
South	71.83	68.08	114.88	47.81

Growth trends were statistically significant through 2020, with subsequent regional variation (Supplementary Table 1). At the national level, detection rates showed a general increasing trend until 2020. For example, among females aged 70–79 years, AAPC 26.56; p-value<0.001; among males aged ≥80 years, AAPC 23.71; p-value<0.001. Between 2020 and 2023, however, most groups remained stable (Supplementary Table 1).

In the Northeast, rates increased across all groups until 2020, after which stability predominated from 2020 to 2023. In the Southeast, growth continued through 2020, but between 2020 and 2023, the pattern persisted in specific subgroups, such as men aged 70–79 years (AAPC 20.63; p-value 0.020), whereas other groups stabilized.

In the Central-West, data showed significant growth until 2020 in all strata, followed by a stable trend. In the North, patterns showed growth until 2020. An exception to the general stabilization pattern was observed among men aged 80 years or older, who continued to show significant growth after 2020 (AAPC 22.37; p-value<0.001).

In the South, data showed growth across all age groups and sexes until 2020. Notably, men aged 70–79 years maintained a growth trend from 2020 to 2023 (AAPC 16.14; p-value 0.008). Other groups showed deceleration, although the trends were not statistically significant.

## Discussion 

The analysis of the results reveals a two-phase epidemiological scenario for syphilis among older adults in Brazil: a period of sharp growth in detection until 2020, followed by stabilization at a plateau with persistently high rates between 2020 and 2023. This dynamic indicates that the infection has become an endemic public health problem in this population. The findings also highlight a marked geographic concentration in the South and Central-West regions. 

This represents a complex global epidemiological context, in which, despite a worldwide decline in syphilis prevalence between 1990 and 2016 (9), more recent evidence from 2020 onward points to a strong resurgence of the disease, characterizing a modern epidemic (10). This new wave of infections has occurred primarily in younger age groups, leading to outbreaks in high-risk populations, such as men who have sex with men (11). There has also been a more recent secondary epidemic among heterosexual men and women, which explains the concerning increase in congenital syphilis (10). Therefore, the data from this study corroborate that Brazil is experiencing this resurgence, further indicating that the older adult population is a critically relevant component of this new epidemiological dynamic (12,13).

This study demonstrated that the increasing trend in detection rates varies across age groups, with older age groups showing greater significance. In China in 2014, a high prevalence of the disease among older adults was also identified (14), and a recent global meta-analysis recognized advanced age as a significant risk factor for syphilis infection (15). The higher contribution of older age groups to syphilis detection rates in Brazil may be associated with their historical exclusion from sexual health policies, a gap that limits opportunities for prevention and treatment, resulting in late diagnoses and severe clinical presentations in this group (16,17,18).

In this analysis, the lack of information on demographic and socioeconomic data in syphilis notifications was identified as a finding that illustrates the difficulty of accurately characterizing the disease in older adults, representing one of the barriers to planning effective policies for this population. This is a limiting factor in assessing how socioeconomic characteristics interact with syphilis risk in older age. In other populations, such as pregnant women, it is known that high prevalence is associated with socioeconomic vulnerability factors, such as low education and Black or Brown race/skin color (19). This association between syphilis and social determinants is a global pattern, with prevalence increasing among populations with lower income and education levels (20,21). Beyond infection risk, socioeconomic barriers, such as limited access to health services, compromise adherence to and completion of treatment, a problem documented globally and in Brazil (22). 

In this analysis, the South and Central-West regions had the highest detection rates, diverging from previous studies that identified the Southeast as more prominent (6,7,18). This shift can be partly explained by the more precise demographic calculation methodology used in this study. From a broader perspective, this regional asymmetry occurs across Latin America, which is one of the regions with the highest prevalence of syphilis among men who have sex with men in the world, reinforcing the importance of monitoring these regional epicenters (10,14). The predominance of cases among males was also evident, a pattern consistent with the literature (6,4). The concentration of cases in these South American regions and groups is alarming, especially given the interaction between syphilis and HIV, where coinfection accelerates the progression of both diseases and increases the risk of HIV transmission (9,23,24,25). 

This study’s evaluation demonstrated a change in trend after 2020, when rapid growth gave way to a plateau at high levels in most groups. This new disease behavior may reflect altered sexual dynamics due to the COVID-19 pandemic, but it may also indicate chronic underreporting of syphilis cases. The pandemic overburdened health systems globally, impacting the capacity for surveillance and notification of other diseases (26). Despite stabilization in most strata, persistent growth was observed in specific subgroups, such as men aged 70–79 years in the South and Southeast and older adults aged 80 years or older in the North, indicating that epidemiological surveillance needs to be intensified in these more vulnerable populations.

It is important to consider how detection rates might vary if official IBGE intercensal projections were adopted. As such, projections underestimated the older adult population in several states relative to the 2022 census, particularly in the South and Central-West regions. Using these values as denominators would yield artificially inflated detection rates. Despite potential numerical variation, exploratory analyses indicated that the overall pattern of growth through 2020, followed by stabilization in the subsequent period, would remain unchanged. Therefore, although different population estimation strategies may slightly alter the absolute values of the rates, the direction of the trends and the main conclusions of this study remain robust despite observed demographic discrepancies.

Gender asymmetry, with higher detection rates among males, is a central finding of this study. Risk behaviors and neglect of condom use, often driven by the mistaken perception of no risk of sexually transmitted infections (STI), may explain this disparity (6,7,27,4). Post-2020 trend analysis, however, reveals that the shift to a plateau of stabilization was common to both sexes in most age groups (Supplementary Table 1). The main exception occurred in the 70–79-year age group, where asymmetry intensified: while females remained stable, males continued to increase. This persistence of growth among older men is concerning, as it increases the risk of severe outcomes and HIV coinfection, amplifying the public health impact. Although infection rates among women are lower, their persistence at elevated levels over time reflects specific vulnerabilities, such as taboos regarding sexuality in older age and lower risk perception, which hinder prevention and diagnosis-seeking behaviors (28,29).

This analysis uses secondary data from SINAN and is subject to limitations, including data availability only through December 2023, underreporting, and inconsistencies (6,7). Additionally, diagnostic accuracy is an important variable. Traditional diagnostic algorithms, which begin with non-treponemal tests, may have reduced sensitivity for primary and latent syphilis cases (30). Global analyses also show that using a single test type (treponemal or nontreponemal) tends to overestimate prevalence relative to the dual-test gold standard (9), complicating the interpretation of surveillance data. 

The underrepresentation of older adults, especially those aged 80 years or older, may result from their exclusion from testing. This practice is based on the perceived absence of risk. This gap limits inferences about infections in advanced age. Moreover, the data used only covers the period up to 31 December 2023, corresponding to the most recent consolidated, audited, and publicly available set of notifications from the Ministry of Health at the time of extraction. Information for 2024 and 2025 was unavailable or under qualification and therefore did not meet the completeness criteria for time series analysis. The absence of data for 2024 and 2025 reflects operational limitations of the surveillance system, not a methodological restriction imposed by the authors. Nonetheless, the study contributes to a better understanding of syphilis among older adults. 

Syphilis detection among older adults in Brazil showed a growth trend until 2020, stabilizing at a plateau of high rates from 2020 to 2023. Higher detection rates were concentrated in the South and Central-West regions, predominated in males, and were especially prominent in the 70–79-year age group. Post-2020 analysis reveals the persistence of growth foci in specific subgroups, indicating ongoing vulnerability. This finding underscores the urgent need to rethink sexual health policies for older adults, as well as targeted prevention and screening strategies to control the spread of syphilis. 

Within the context of the Brazilian Unified Health System (*Sistema Único de Saúde*, SUS), this growth trend may indicate similar risks for other sexually transmitted infections, such as HIV, highlighting the importance of strengthening integrated surveillance. To address this scenario, it is essential to invest in the training of primary health care professionals, develop educational campaigns specific to sexuality in older age, and expand access to rapid testing and timely treatment at primary health care units. 

Consequently, it would be appropriate to establish sexual health care pathways for older adults, integrated into health care networks, and to incorporate STI testing whenever indicated. Future research should explore the social and behavioral determinants of the acceleration in rates in the post-pandemic period and evaluate the effectiveness of targeted interventions within SUS. Furthermore, research should expand to more clearly assess the impact of syphilis on the health of older adults from a clinical perspective within SUS, which would require broader data collection to evaluate cure rates and treatment effectiveness more meaningfully.

## Data Availability

The replication dataset supporting the findings of this study is publicly available in the SciELO Data repository under the Digital Object Identifier (DOI): https://doi.org/10.48331/SCIELODATA.NQ5H7D. Epidemiological data on syphilis can also be accessed through the Department of Informatics of the Brazilian Unified Health System Information Technology Department ( Departamento de Informática do Sistema Único de Saúde, DATASUS) portal, in the Notifiable Diseases Information System (Sistema de Informação de Agravos de Notificação, SINAN) section: http://www.datasus.gov.br. Demographic data can be consulted on the Brazilian Institute of Geography and Statistics (Instituto Brasileiro de Geografia e Estatística, IBGE) portal at: https://www.ibge.gov.br.
