# Supplementary material for: Trend in the detection rate of syphilis among older adults: time series analysis, Brazil, 2014–2023
Source: Epidemiol Serv Saude. 2026 Apr 10;35:e20250900. doi: 10.1590/S2237-96222026v35e20250900.en (PMC13078671; doi:10.1590/S2237-96222026v35e20250900.en)
Supplement: Supplementary file 1 [file 2237-9622-ress-35-e20250900-supp01-en.pdf]

Supplementary Table 1. Adjusted average annual percent change (AAPC), statistical significance (p-value), and 95% confidence interval (95%CI) of syphilis detection rates among older adults, by sex, age group, and macroregion. Brazil, 2014–2023

Region	Sex	Age group (years)	AAPC 2014–2020	p-value	95%CI	Trend	AAPC 2020–2023	p-value	95%CI	Trend
**Brazil**	Female	60–64	17.70	<0.001	12.49; 27.69	Increase	-2.27	0.508	-18.69; 5.85	Stability
**Brazil**	Female	65–69	17.54	<0.001	12.91; 25.44	Increase	-0.86	0.746	-14.79; 6.46	Stability
**Brazil**	Female	70–79	26.56	<0.001	22.31; 32.84	Increase	1.25	0.797	-8.72; 8.17	Stability
**Brazil**	Female	80+	20.90	<0.001	18.19; 24.54	Increase	0.98	0.729	-4.76; 5.71	Stability
**Brazil**	Male	60–64	17.10	<0.001	13.25; 23.73	Increase	-0.69	0.717	-12.28; 5.82	Stability
**Brazil**	Male	65–69	20.00	<0.001	15.79; 27.19	Increase	-1.73	0.503	-13.16; 5.29	Stability
**Brazil**	Male	70–79	17.99	0.022	2.35; 35.79	Increase	17.99	0.022	2.35; 35.79	Increase
**Brazil**	Male	80+	23.71	<0.001	21.76; 26.21	Increase	3.44	0.050	0.01; 6.75	Stability
**Central-West**	Female	60–64	23.96	<0.001	19.78; 29.91	Increase	-4.31	0.209	-12.90; 2.72	Stability
**Central-West**	Female	65–69	26.09	<0.001	21.09; 34.00	Increase	-2.81	0.414	-14.51; 5.47	Stability
**Central-West**	Female	70–79	39.43	<0.001	31.56; 49.99	Increase	0.44	0.974	-15.40; 11.95	Stability
**Central-West**	Female	80+	38.09	<0.001	27.18; 57.12	Increase	-5.32	0.431	-29.61; 10.01	Stability
**Central-West**	Male	60–64	20.28	<0.001	15.44; 28.29	Increase	-1.83	0.575	-15.63; 6.18	Stability
**Central-West**	Male	65–69	27.01	<0.001	21.82; 36.52	Increase	0.43	0.971	-8.60; 7.39	Stability
**Central-West**	Male	70–79	40.88	<0.001	21.39; 69.59	Increase	7.04	0.705	-27.86; 23.78	Stability
**Central-West**	Male	80+	30.70	<0.001	24.42; 40.88	Increase	-3.45	0.396	-16.94; 6.25	Stability
**Northeast**	Female	60–64	35.34	<0.001	25.68; 54.26	Increase	4.28	0.631	-23.77; 18.21	Stability
**Northeast**	Female	65–69	37.62	<0.001	29.80; 49.67	Increase	7.24	0.440	-11.70; 19.33	Stability
**Northeast**	Female	70–79	47.90	<0.001	36.23; 69.16	Increase	10.77	0.330	-21.64; 26.59	Stability
**Northeast**	Female	80+	53.36	<0.001	45.98; 65.69	Increase	11.34	0.102	-4.65; 22.52	Stability
**Northeast**	Male	60–64	37.52	<0.001	30.79; 48.69	Increase	6.49	0.361	-10.27; 17.00	Stability
**Northeast**	Male	65–69	39.74	<0.001	30.98; 54.72	Increase	2.54	0.793	-17.84; 16.00	Stability
**Northeast**	Male	70–79	49.29	0.006	11.18; 102.76	Increase	16.00	0.370	-43.46; 74.94	Stability
**Northeast**	Male	80+	48.61	<0.001	41.34; 62.19	Increase	14.38	0.098	-5.80; 25.85	Stability
**North**	Female	60–64	28.97	<0.001	22.50; 39.38	Increase	2.01	0.755	-15.53; 11.76	Stability
**North**	Female	65–69	16.02	0.031	1.58; 32.26	Increase	16.02	0.031	1.58; 32.26	Increase
**North**	Female	70–79	38.80	<0.001	28.55; 56.32	Increase	3.20	0.772	-24.00; 17.84	Stability
**North**	Female	80+	38.28	<0.001	31.30; 48.82	Increase	5.58	0.376	-10.64; 16.39	Stability
**North**	Male	60–64	28.75	<0.001	22.92; 40.90	Increase	5.00	0.288	-8.11; 12.40	Stability

Region	Sex	Age group (years)	AAPC 2014–2020	p-value	95%CI	Trend	AAPC 2020–2023	p-value	95%CI	Trend
**North**	Male	65–69	28.91	<0.001	22.50; 38.73	Increase	3.16	0.696	-12.18; 13.37	Stability
**North**	Male	70–79	28.77	<0.001	10.08; 50.33	Increase	28.77	<0.001	10.08; 50.33	Increase
**North**	Male	80+	22.37	<0.001	15.86; 29.25	Increase	22.37	<0.001	15.86; 29.25	Increase
**Southeast**	Female	60–64	38.48	<0.001	25.89; 58.47	Increase	6.75	0.625	-23.60; 22.61	Stability
**Southeast**	Female	65–69	40.17	<0.001	32.01; 51.75	Increase	10.91	0.222	-8.96; 22.30	Stability
**Southeast**	Female	70–79	62.39	<0.001	56.61; 70.29	Increase	4.01	0.243	-3.05; 11.79	Stability
**Southeast**	Female	80+	77.23	0.002	30.23; 147.47	Increase	24.79	0.316	-42.32; 91.97	Stability
**Southeast**	Male	60–64	38.02	<0.001	32.05; 45.78	Increase	3.02	0.622	-8.63; 12.08	Stability
**Southeast**	Male	65–69	42.33	<0.001	34.16; 56.71	Increase	13.47	0.100	-4.49; 23.06	Stability
**Southeast**	Male	70–79	69.75	<0.001	53.90; 91.26	Increase	20.63	0.020	3.62; 32.09	Increase
**Southeast**	Male	80+	48.88	<0.001	39.53; 65.16	Increase	10.35	0.272	-14.17; 24.35	Stability
**South**	Female	60–64	16.13	<0.001	9.02; 29.68	Increase	-1.11	0.656	-22.01; 6.63	Stability
**South**	Female	65–69	12.17	<0.001	6.23; 22.31	Increase	-1.67	0.613	-19.62; 7.85	Stability
**South**	Female	70–79	20.23	<0.001	15.28; 29.67	Increase	6.32	0.227	-9.94; 12.04	Stability
**South**	Female	80+	17.79	<0.001	11.77; 26.66	Increase	3.24	0.568	-8.88; 8.36	Stability
**South**	Male	60–64	16.42	<0.001	12.10; 24.42	Increase	1.80	0.683	-10.14; 7.10	Stability
**South**	Male	65–69	17.36	<0.001	12.52; 26.61	Increase	-1.82	0.566	-17.07; 6.05	Stability
**South**	Male	70–79	16.14	0.008	3.69; 30.13	Increase	16.14	0.008	3.69; 30.13	Increase
**South**	Male	80+	18.77	<0.001	14.78; 26.15	Increase	5.42	0.194	-6.58; 10.30	Stability

Supplementary Figure 1 – Temporal variation in the syphilis detection rate among older adults by region

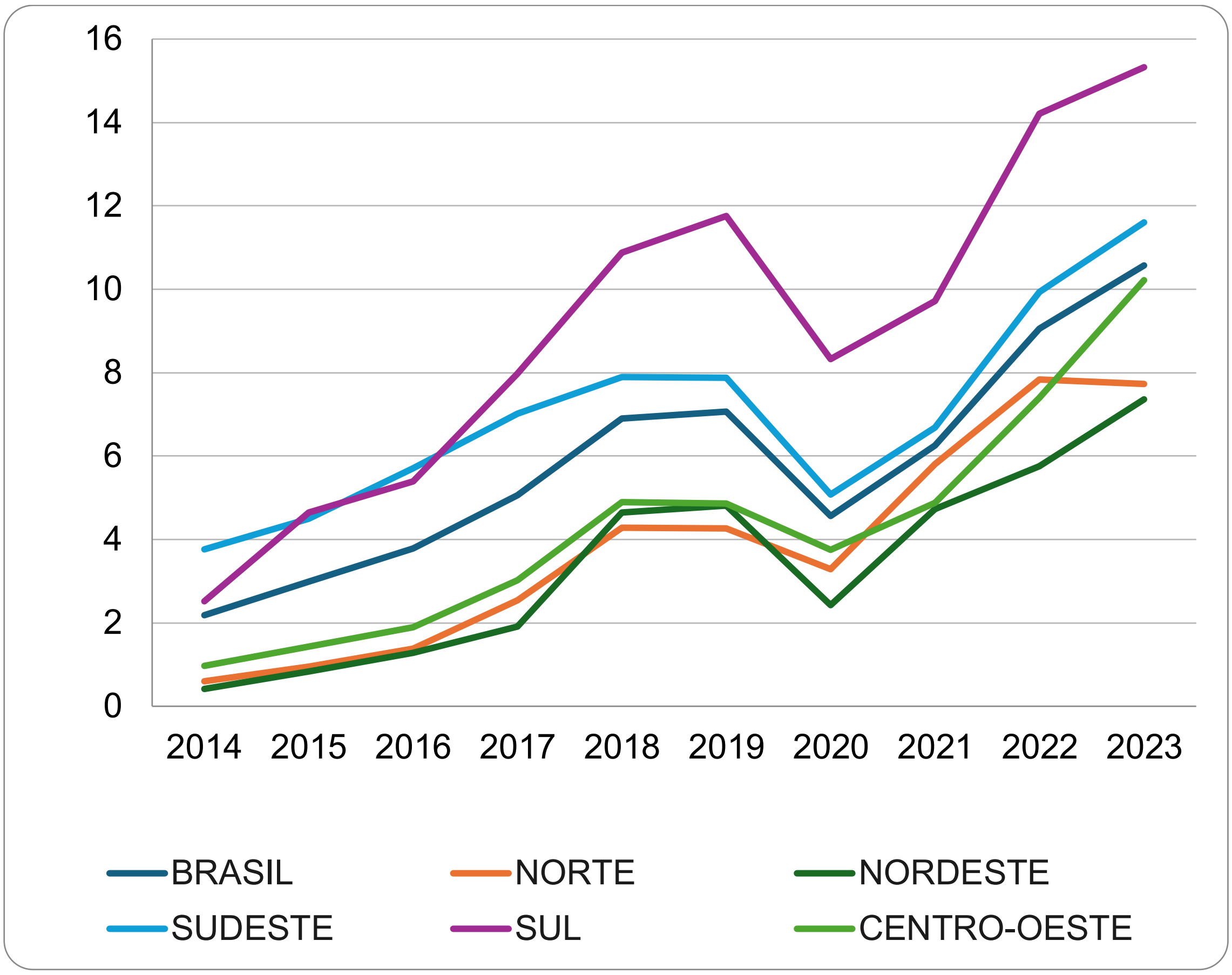

BRAZIL	NORTH	NORTHEAST
SOUTHEAST	SOUTH	CENTRAL-WEST
