# Supplementary material for: Trend in the detection rate of syphilis among older adults: time series analysis, Brazil, 2014–2023
Source: Epidemiol Serv Saude. 2026 Apr 10;35:e20250900. doi: 10.1590/S2237-96222026v35e20250900.en (PMC13078671; doi:10.1590/S2237-96222026v35e20250900.en)

Tabela Suplementar 1: Taxa média anual de variação percentual ajustada (AAPC), medida de significância (p-valor) e intervalo de confiança de 95% (IC95%) na detecção de sífilis em pessoas idosas por sexo, faixa etária e macrorregião. Brasil, 2014-2023

Região	Sexo	Faixa etária (anos)	AAPC 2014-2020	p-valor	IC95%	Tendência	AAPC 2020-2023	p-valor	IC95%	Tendência
**Brasil**	Feminino	60-64	17,70	<0,001	12,49; 27,69	Crescimento	-2,27	0,508	-18,69; 5,85	Estabilidade
**Brasil**	Feminino	65-69	17,54	<0,001	12,91; 25,44	Crescimento	-0,86	0,746	-14,79; 6,46	Estabilidade
**Brasil**	Feminino	70-79	26,56	<0,001	22,31; 32,84	Crescimento	1,25	0,797	-8,72; 8,17	Estabilidade
**Brasil**	Feminino	80+	20,90	<0,001	18,19; 24,54	Crescimento	0,98	0,729	-4,76; 5,71	Estabilidade
**Brasil**	Masculino	60-64	17,10	<0,001	13,25; 23,73	Crescimento	-0,69	0,717	-12,28; 5,82	Estabilidade
**Brasil**	Masculino	65-69	20,00	<0,001	15,79; 27,19	Crescimento	-1,73	0,503	-13,16; 5,29	Estabilidade
**Brasil**	Masculino	70-79	17,99	0,022	2,35; 35,79	Crescimento	17,99	0,022	2,35; 35,79	Crescimento
**Brasil**	Masculino	80+	23,71	<0,001	21,76; 26,21	Crescimento	3,44	0,050	0,01; 6,75	Estabilidade
**Centro-Oeste**	Feminino	60-64	23,96	<0,001	19,78; 29,91	Crescimento	-4,31	0,209	-12,90; 2,72	Estabilidade
**Centro-Oeste**	Feminino	65-69	26,09	<0,001	21,09; 34,00	Crescimento	-2,81	0,414	-14,51; 5,47	Estabilidade
**Centro-Oeste**	Feminino	70-79	39,43	<0,001	31,56; 49,99	Crescimento	0,44	0,974	-15,40; 11,95	Estabilidade
**Centro-Oeste**	Feminino	80+	38,09	<0,001	27,18; 57,12	Crescimento	-5,32	0,431	-29,61; 10,01	Estabilidade
**Centro-Oeste**	Masculino	60-64	20,28	<0,001	15,44; 28,29	Crescimento	-1,83	0,575	-15,63; 6,18	Estabilidade
**Centro-Oeste**	Masculino	65-69	27,01	<0,001	21,82; 36,52	Crescimento	0,43	0,971	-8,60; 7,39	Estabilidade
**Centro-Oeste**	Masculino	70-79	40,88	<0,001	21,39; 69,59	Crescimento	7,04	0,705	-27,86; 23,78	Estabilidade
**Centro-Oeste**	Masculino	80+	30,70	<0,001	24,42; 40,88	Crescimento	-3,45	0,396	-16,94; 6,25	Estabilidade
**Nordeste**	Feminino	60-64	35,34	<0,001	25,68; 54,26	Crescimento	4,28	0,631	-23,77; 18,21	Estabilidade
**Nordeste**	Feminino	65-69	37,62	<0,001	29,80; 49,67	Crescimento	7,24	0,440	-11,70; 19,33	Estabilidade
**Nordeste**	Feminino	70-79	47,90	<0,001	36,23; 69,16	Crescimento	10,77	0,330	-21,64; 26,59	Estabilidade
**Nordeste**	Feminino	80+	53,36	<0,001	45,98; 65,69	Crescimento	11,34	0,102	-4,65; 22,52	Estabilidade
**Nordeste**	Masculino	60-64	37,52	<0,001	30,79; 48,69	Crescimento	6,49	0,361	-10,27; 17,00	Estabilidade
**Nordeste**	Masculino	65-69	39,74	<0,001	30,98; 54,72	Crescimento	2,54	0,793	-17,84; 16,00	Estabilidade
**Nordeste**	Masculino	70-79	49,29	0,006	11,18; 102,76	Crescimento	16,00	0,370	-43,46; 74,94	Estabilidade
**Nordeste**	Masculino	80+	48,61	<0,001	41,34; 62,19	Crescimento	14,38	0,098	-5,80; 25,85	Estabilidade
**Norte**	Feminino	60-64	28,97	<0,001	22,50; 39,38	Crescimento	2,01	0,755	-15,53; 11,76	Estabilidade
**Norte**	Feminino	65-69	16,02	0,031	1,58; 32,26	Crescimento	16,02	0,031	1,58; 32,26	Crescimento
**Norte**	Feminino	70-79	38,80	<0,001	28,55; 56,32	Crescimento	3,20	0,772	-24,00; 17,84	Estabilidade
**Norte**	Feminino	80+	38,28	<0,001	31,30; 48,82	Crescimento	5,58	0,376	-10,64; 16,39	Estabilidade
**Norte**	Masculino	60-64	28,75	<0,001	22,92; 40,90	Crescimento	5,00	0,288	-8,11; 12,40	Estabilidade

Região	Sexo	Faixa etária (anos)	AAPC 2014-2020	p-valor	IC95%	Tendência	AAPC 2020-2023	p-valor	IC95%	Tendência
**Norte**	Masculino	65-69	28,91	<0,001	22,50; 38,73	Crescimento	3,16	0,696	-12,18; 13,37	Estabilidade
**Norte**	Masculino	70-79	28,77	<0,001	10,08; 50,33	Crescimento	28,77	<0,001	10,08; 50,33	Crescimento
**Norte**	Masculino	80+	22,37	<0,001	15,86; 29,25	Crescimento	22,37	<0,001	15,86; 29,25	Crescimento
**Sudeste**	Feminino	60-64	38,48	<0,001	25,89; 58,47	Crescimento	6,75	0,625	-23,60; 22,61	Estabilidade
**Sudeste**	Feminino	65-69	40,17	<0,001	32,01; 51,75	Crescimento	10,91	0,222	-8,96; 22,30	Estabilidade
**Sudeste**	Feminino	70-79	62,39	<0,001	56,61; 70,29	Crescimento	4,01	0,243	-3,05; 11,79	Estabilidade
**Sudeste**	Feminino	80+	77,23	0,002	30,23; 147,47	Crescimento	24,79	0,316	-42,32; 91,97	Estabilidade
**Sudeste**	Masculino	60-64	38,02	<0,001	32,05; 45,78	Crescimento	3,02	0,622	-8,63; 12,08	Estabilidade
**Sudeste**	Masculino	65-69	42,33	<0,001	34,16; 56,71	Crescimento	13,47	0,100	-4,49; 23,06	Estabilidade
**Sudeste**	Masculino	70-79	69,75	<0,001	53,90; 91,26	Crescimento	20,63	0,020	3,62; 32,09	Crescimento
**Sudeste**	Masculino	80+	48,88	<0,001	39,53; 65,16	Crescimento	10,35	0,272	-14,17; 24,35	Estabilidade
**Sul**	Feminino	60-64	16,13	<0,001	9,02; 29,68	Crescimento	-1,11	0,656	-22,01; 6,63	Estabilidade
**Sul**	Feminino	65-69	12,17	<0,001	6,23; 22,31	Crescimento	-1,67	0,613	-19,62; 7,85	Estabilidade
**Sul**	Feminino	70-79	20,23	<0,001	15,28; 29,67	Crescimento	6,32	0,227	-9,94; 12,04	Estabilidade
**Sul**	Feminino	80+	17,79	<0,001	11,77; 26,66	Crescimento	3,24	0,568	-8,88; 8,36	Estabilidade
**Sul**	Masculino	60-64	16,42	<0,001	12,10; 24,42	Crescimento	1,80	0,683	-10,14; 7,10	Estabilidade
**Sul**	Masculino	65-69	17,36	<0,001	12,52; 26,61	Crescimento	-1,82	0,566	-17,07; 6,05	Estabilidade
**Sul**	Masculino	70-79	16,14	0,008	3,69; 30,13	Crescimento	16,14	0,008	3,69; 30,13	Crescimento
**Sul**	Masculino	80+	18,77	<0,001	14,78; 26,15	Crescimento	5,42	0,194	-6,58; 10,30	Estabilidade

Figura Suplementar 1 – Variação temporal da taxa de detecção de sífilis em pessoas idosas por região

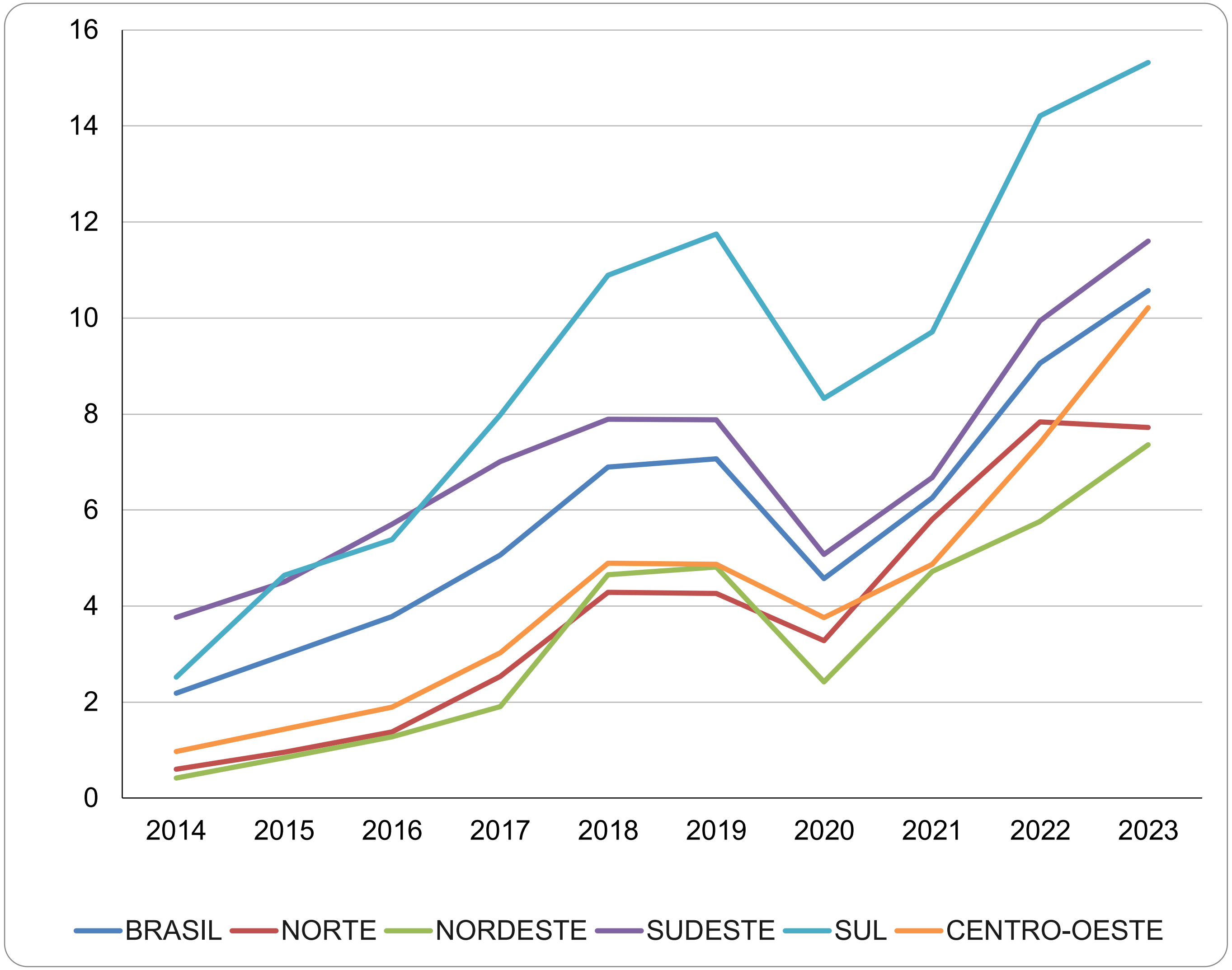

Supplement: Supplementary file 2 [file 2237-9622-ress-35-e20250900-supp01-pt.pdf]
