# Supplementary material for: Trend in the detection rate of syphilis among older adults: time series analysis, Brazil, 2014–2023
Source: Epidemiol Serv Saude. 2026 Apr 10;35:e20250900. doi: 10.1590/S2237-96222026v35e20250900.en (PMC13078671; doi:10.1590/S2237-96222026v35e20250900.en)
Supplement: Translation

# Translation: Tendência da taxa de detecção de sífilis em idosos: análise de série temporal, Brasil, 2014-2023

**DOI:** 10.1590/S2237-96222026v35e20250900.pt

**Authors:** Gustavo Gabriel Oliveira, Anderson da Silveira Gonçalves, Heitor Wermann, Renan Garcia Artus, Felipe Groppelli, Nathiele Nunes da Silva Gonçalves

**Affiliations:** 1 Universidade Federal de Ciências da Saúde de Porto Alegre, Porto Alegre, RS, Brasil; 2 Atitus Educação, Porto Alegre, RS, Brasil

**Keywords:** Pessoas Idosas, Sífilis, Envelhecimento, Vigilância em Saúde Pública, Estudos de Séries Temporais

## Abstract

**Objetivo:**

Analisar a tendência de detecção de sífilis em pessoas idosas no Brasil no período de 2014 a 2023.

**Métodos:**

Estudo analítico de séries temporais com dados de sífilis adquirida em indivíduos com 60 anos ou mais, obtidos do Sistema de Informação de Agravos de Notificação. A análise de tendência foi realizada por regressão segmentada, calculando a Variação Percentual Anual Média (Average Annual Percent Change, AAPC). Foram analisadas variáveis de sexo, faixa etária e macrorregião.

**Resultados:**

Foram notificados 117.297 casos (8,28% das notificações totais). A taxa média de detecção foi 59,80 casos por 100 mil pessoas idosas, maior em homens (77,39/100 mil contra 50,65/100 mil em mulheres). As maiores taxas ocorreram na faixa 70-79 anos (80,75/100 mil), particularmente nas regiões Sul (114,88/100 mil) e Centro-Oeste (102,68/100 mil). Até 2020, verificou-se tendência nacional de crescimento significativo (em mulheres 70-79 anos, AAPC 26,56; homens ≥80 anos, AAPC 23,71). Entre 2020 e 2023, a maioria dos grupos apresentou estabilidade, embora persistisse aumentos em subgrupos, como homens de 70-79 anos no Sudeste (AAPC 20,63) e no Sul (AAPC 16,14).

**Conclusão:**

A sífilis avançou significativamente até 2020 e estabilizou-se em um platô elevado. As disparidades de gênero e regionais foram evidentes: as taxas médias foram maiores em homens do que em mulheres e nas regiões Sul e Centro-Oeste em comparação à média nacional. O crescimento persistente após 2020 evidencia um problema de saúde pública demandando políticas de saúde sexual direcionadas.

**Aspectos éticos**

Esta pesquisa utilizou bancos de dados de domínio público e anonimizados.:

## Introdução 

O envelhecimento da população brasileira aumentou nas últimas décadas, evidenciando uma transformação demográfica no país. O número de pessoas com 60 anos ou mais cresceu, refletindo o aumento da expectativa de vida (1). Esse fenômeno apresenta desafios à adaptação da realidade das políticas públicas de saúde, demandando mudanças que visem garantir qualidade de vida a essa parcela da população. Esse crescimento da população idosa pode ser atribuído a diversos fatores como a queda na mortalidade, avanços na medicina e melhoria na atenção primária à saúde. 

Os dados indicam que essa tendência de crescimento populacional deve se manter nos próximos anos, pressionando sistemas de saúde e de previdência social, que precisarão se adaptar para atender às novas demandas de uma população com uma variedade de doenças crônicas, agudas, incapacidades e necessidades de cuidados especializados (2). O aumento da população idosa acompanha o crescimento das infecções por sífilis, pois o desconhecimento dessa população acerca das infecções sexualmente transmissíveis (IST), somado à comportamentos de risco, predispõe pessoas idosas à contaminação (3,4). Apesar disso, poucos estudos incluem esse grupo para avaliar a temática (5). 

No Brasil, a tendência crescente da taxa de detecção de sífilis entre pessoas idosas foi identificada no intervalo de 2011 a 2019 (6). Em 2020, a prevalência de sífilis foi estimada e demonstrou-se uma taxa de detecção de 12,84 casos por 100 mil brasileiros, com destaque para homens nas regiões Sul e Sudeste (7). Entretanto, estudos ainda não compararam dados antes e após a pandemia de covid-19 para analisar os efeitos deste evento nas tendências de sífilis, o que limita a entendimento do panorama no Brasil, justificando-se, portanto, a proposta deste estudo. A pandemia pode ter alterado os comportamentos sexuais das pessoas idosas, bem como no diagnóstico e tratamento da sífilis. Por isso, avaliar os períodos com enfoque na covid-19 é fundamental para verificar se as tendências crescentes previamente observadas se mantiveram, se houve mudanças regionais ou de perfil populacional entre as pessoas idosas, objeto deste estudo. 

## Métodos 

### Delineamento 

Trata-se de um estudo analítico de séries temporais, conduzido com o objetivo de analisar a tendência da taxa de detecção de sífilis em pessoas idosas (60 anos ou mais) no Brasil, no período de 2014 a 2023. As unidades de análise foram desagregadas em nível subnacional (27 unidades federativas, incluindo o Distrito Federal) e macrorregional (Norte, Nordeste, Sudeste, Sul e Centro-Oeste) conforme os critérios definidos pelo Instituto Brasileiro de Geografia e Estatística (IBGE). Como todos os registros disponíveis do Sistema de Informação de Agravos de Notificação (SINAN) foram incluídos, não se aplicou cálculo de tamanho amostral, uma vez que a análise foi conduzida sobre o universo completo de notificações, caracterizando uma abordagem da totalidade de dados disponíveis para o período em estudo. Essa estratégia maximiza a abrangência e o poder estatístico da análise e fornece um panorama nacional do agravo com base em dados oficiais de vigilância epidemiológica. O conjunto de dados final gerado para esta análise, incluindo os casos compilados e as projeções populacionais, foi depositado em repositório público (8).

### Fonte de dados

Os dados epidemiológicos de sífilis adquirida em indivíduos com 60 anos ou mais foram obtidos do SINAN, sistema gerenciado pelo Ministério da Saúde e alimentado pelos serviços de saúde municipais e estaduais. A notificação de sífilis é compulsória, realizada por profissionais de saúde (técnicos em enfermagem, enfermeiros e médicos) capacitados e responsáveis pelo diagnóstico e atendimento do paciente. Notifica-se por meio da Ficha Individual de Notificação e da Ficha de Investigação, ambas padronizadas pelo Ministério da Saúde, em atendimentos de todos os níveis de atenção à saúde. As informações são inseridas nos sistemas informatizados e-SUS/SinanNet e consolidadas pelas secretarias municipais, estaduais, distrital e pelo Ministério da Saúde. A definição de caso seguiu os critérios do Guia de Vigilância em Saúde, editado pelo Ministério da Saúde em 2023. Considerou-se a detecção de anticorpos treponêmicos (teste rápido ou teste laboratorial) associada à confirmação por teste não treponêmico com titulação significativa. 

### População em estudo

A população do estudo compreende pessoas idosas (≥60 anos) notificadas no SINAN entre 1º de janeiro de 2014 e 31 de dezembro de 2023. As análises foram conduzidas considerando o local de residência do caso notificado, respeitando as divisões estaduais e macrorregionais do Brasil. Foram incluídas todas as notificações confirmadas de sífilis adquirida em pessoas com 60 anos ou mais. Excluíram-se notificações com diagnóstico negativo, inconclusivo ou com inconsistências no campo “faixa etária”, como fichas de notificação com esse campo em branco.

### Variáveis 

A variável dependente foi a taxa de detecção de sífilis adquirida em pessoas idosas. As variáveis independentes incluíram: sexo (masculino e feminino); faixa etária (em anos, 60-64, 65-69, 70-79 e ≥80); raça/cor da pele (branca, amarela, parda, indígena e preta); escolaridade (analfabeto, ensino fundamental, médio e superior); região de notificação (Norte, Nordeste, Sudeste, Sul e Centro-Oeste); desfecho do caso (cura ou óbito por sífilis ou outra causa). Os dados ignorados ou não informados foram mantidos e classificados na categoria “ignorado”, o que permitiu avaliação da completude e minimizou perda de informação.

### Estimativas populacionais e cálculo das taxas

As estimativas populacionais para o denominador das taxas de detecção foram obtidas a partir dos censos demográficos de 2010 e 2022 do IBGE por meio dos valores disponibilizados em cada faixa etária mencionada anteriormente, por sexo e estado da federação. Para os anos intercensitários (2014-2021), aplicou-se interpolação exponencial e, para 2023, extrapolação sobre os valores censitários de 2022, com base na taxa de crescimento geométrico anual média (r), calculada por sexo e faixa etária em cada unidade federativa segundo a fórmula: r=((P2022/P2010)^(1/12)) - 1. A projeção populacional de cada estado foi estimada conforme: Pt=P0×(1+r)^t, em que P0 é a população do censo de 2010 e t o número de anos transcorridos. Esse método foi adotado para garantir coerência entre as duas contagens censitárias, uma vez que estimativas intercensitárias do IBGE, realizadas em 2018, apresentaram discrepâncias relevantes quando comparadas ao censo de 2022. Tais diferenças podem ser explicadas por alterações na previsão do órgão sobre migrações, as quais foram afetadas principalmente pela pandemia de covid-19 entre os anos 2019 e 2022. Também houve variações nos valores encontrados entre as expectativas de vida regionais e a real longevidade populacional. A escolha foi justificada pela busca de maior estabilidade e alinhamento à tendência demográfica observada. As taxas de detecção foram expressas por 100 mil pessoas idosas.

### Análise estatística e de tendência temporal

A tendência temporal das taxas de detecção foi avaliada por regressão segmentada (Joinpoint Regression Program, versão 5.4.0). O modelo identifica pontos de inflexão significativos e estima as variações percentuais anuais (*Annual Percent Change*, APC) e variação percentual anual média (*Average Annual Percent Change*, AAPC), com intervalos de confiança de 95% (IC95%) e p-valor<0,05. Não se consideraram outras abordagens comparativas, a exemplo da Média Móvel Integrada Autorregressiva (*AutoRegressive Integrated Moving Average*, ARIMA) ou do Sistema Geral de Modelagem Algébrica General (*Algebraic Modeling System*, GAMS), porque a regressão segmentada é melhor para detectar a APC e é o único método que identifica estatisticamente a mudança de tendência, calculando uma APC distinta e fácil de interpretar para cada período linear identificado. 

Foi pré-especificado um ponto de inflexão em 2020, com justificativa epidemiológica baseada no impacto da pandemia de covid-19 sobre a vigilância sanitária e o diagnóstico da sífilis. A escolha desse ponto permitiu comparar as tendências em dois segmentos: 2014-2020 e 2020-2023. Não foi necessário gerar outros pontos de inflexão na análise, tendo em vista que houve crescimento linear nos demais períodos. A função pesquisa em grade com teste de permutação do programa foi utilizada para avaliar se a inclusão do ponto fixo aprimorava o ajuste do modelo, o que se confirmou. Foram avaliados os pressupostos de independência dos resíduos, homocedasticidade e ausência de autocorrelação temporal. Análises de sensibilidade foram conduzidas para verificar possíveis influências de valores extremos e a robustez das estimativas em curtos intervalos de tempo dentro do período 2020-2023. A classificação final da tendência considerou os resultados: “crescente” a AAPC>0 e p-valor<0,05; “decrescente” a AAPC<0 e p-valor<0,05 e “estável” p-valor≥0,05. As análises e a organização dos bancos de dados foram realizadas no Microsoft Excel e no Joinpoint Regression Program, o que garantiu reprodutibilidade e transparência metodológica conforme as diretrizes Fortalecimento do Relato de Estudos Observacionais em Epidemiologia (*Strengthening the Reporting of Observational studies in Epidemiology*, STROBE) e Relato de estudos conduzidos usando dados observacionais e registros (*REporting of studies Conducted using Observational Data e registros*, RECORD). 

## Resultados 

No período de 2014 a 2023, foram notificados 117.297 casos de sífilis adquirida em pessoas idosas (60 anos ou mais), correspondendo a 8,28% do total de notificações no Brasil (n=1.415.531).

A média geral da taxa de detecção no período analisado foi de 59,80 casos/100 mil habitantes, sendo superior no sexo masculino (77,39/100 mil). A idade média no momento da notificação foi de 68,38 anos (± 6,^5^). O grupo etário de 60-64 anos foi o mais representativo (38,4% dos homens; 38,23% das mulheres), seguido pela faixa de 65-69 anos (26,99% dos homens; 25,93% das mulheres).

Quanto às variáveis sociodemográficas, observou-se uma quantidade significativa de dados incompletos. Em relação à raça/cor, 14,69% das notificações não continham essa informação; entre as preenchidas, 40,57% dos indivíduos foram declarados como brancos e 33,23% como pardos. Para a escolaridade, 41,20% dos registros estavam em branco; dos preenchidos, 14,36% apontaram ensino fundamental incompleto e 2,42% ensino superior completo.

O desfecho clínico não foi informado em 50,46% das notificações. Dos casos em que havia tal informação, 48,98% evoluíram para cura. Os óbitos atribuídos diretamente à sífilis representaram 0,08% dos casos, e aqueles decorrentes de outras causas, 0,46%.

As regiões Sul e Centro-Oeste apresentaram taxas de detecção superiores à média nacional (Tabela 1 e Figura Suplementar 1). A região Sul registrou taxas de 71,83/100 mil habitantes (60-64 anos) e 68,08/100 mil (65-69 anos), enquanto a média brasileira foi de 60,65 e 57,01, respectivamente. Na região Centro-Oeste, as taxas foram de 60,25 (60-64 anos) e 55,27 (65-69 anos). O pico de detecção ocorreu na faixa de 70-79 anos, em que as taxas regionais também superaram a média brasileira (80,75). Na faixa de 80 anos ou mais, os valores no Sul (47,81) e Centro-Oeste (44,79) permaneceram acima da média nacional (40,79).

**Tabela 1:** Taxa de detecção média (por 100 mil pessoas idosas), por região e faixa etária. Brasil, 2014-2023 (n=32281479)

Região	60-64 anos	65-69 anos	70-79 anos	80+ anos
Brasil	60,65	57,01	80,75	40,79
Centro-Oeste	60,25	55,27	102,68	44,79
Nordeste	44,81	42,91	74,45	28,15
Norte	69,57	62,39	106,75	43,18
Sudeste	56,12	52,43	74,86	36,63
Sul	71,83	68,08	114,88	47,81

Observou-se que as tendências de crescimento da doença foram estatisticamente significativas até 2020, com variações posteriores entre as regiões (Tabela Suplementar 1). Nacionalmente, as taxas de detecção apresentaram tendência geral de crescimento até 2020, Por exemplo, para indivíduos do sexo feminino, na faixa etária 70-79 anos, AAPC 26,56; p-valor<0,001; indivíduos do sexo masculino, com 80+ anos, AAPC 23,71; p-valor<0,001. Entre 2020 e 2023, no entanto, a maioria dos grupos apresentou estabilidade (Tabela Suplementar 1).

No Nordeste, verificou-se crescimento das taxas em todos os grupos até 2020, seguido por predominância de estabilidade no período 2020-2023. No Sudeste, também houve tendência de crescimento até 2020, mas entre 2020 e 2023, o padrão de crescimento manteve-se em subgrupos específicos, como homens de 70 a 79 anos (AAPC 20,63; p-valor 0,020), enquanto os demais grupos passaram a apresentar estabilidade.

Na região Centro-Oeste, os dados apontaram crescimento significativo até 2020 em todos os estratos, seguido por uma tendência de estabilidade. No Norte, os padrões mostraram crescimento até 2020. Uma exceção ao padrão geral de estabilização foi observada no grupo de homens com 80 anos ou mais, que manteve crescimento significativo após 2020 (AAPC 22,37; p-valor<0,001).

No Sul, os dados demonstraram crescimento até 2020 em todas as faixas etárias e sexos. Destaca-se o grupo de homens de 70 a 79 anos, que manteve tendência de crescimento de 2020 a 2023 (AAPC 16,14; p-valor 0,008). Os demais grupos apresentaram desaceleração, com tendências não significativas.

## Discussão 

A análise dos resultados revela um cenário epidemiológico de duas fases para a sífilis em pessoas idosas no Brasil: um período de crescimento acentuado na detecção até 2020, seguido pela estabilização em um platô com taxas persistentemente elevadas entre 2020 e 2023. Essa dinâmica indica que a infecção se consolidou como um problema de saúde pública endêmico nessa população. Os achados também expõem uma concentração geográfica marcante nas regiões Sul e Centro-Oeste. 

Trata-se de um contexto epidemiológico global complexo, no qual, ainda que tenha havido declínio no mundo todo da prevalência de sífilis entre 1990 e 2016 (9), evidências mais recentes, a partir de 2020, apontam para uma forte ressurgência da doença, caracterizando uma epidemia moderna (10). Essa nova onda de infecções tem acontecido em faixas etárias mais jovens, promovendo surtos em populações de alto risco, como homens que fazem sexo com homens (11). Também tem havido uma epidemia secundária mais recente entre homens e mulheres heterossexuais, o que explica o aumento preocupante da sífilis congênita (10). Assim, os dados deste estudo corroboram que o Brasil acompanha essa tendência de ressurgência, acrescentando que a população idosa é um componente crítico relevante dessa nova dinâmica epidemiológica (12,13).

Demonstrou-se neste estudo que a tendência crescente das taxas de detecção apresenta diferenças entre as faixas etárias analisadas, por exemplo maior significância de grupos etários mais velhos. Na China em 2014, identificou-se também uma alta prevalência da doença entre adultos mais velhos (14), e uma recente meta-análise global identificou a idade avançada como um fator de risco significativo para a infecção por sífilis (15). A maior participação de faixas etárias mais velhas nas taxas de detecção da sífilis no Brasil pode estar associada à sua histórica exclusão das políticas de saúde sexual, uma lacuna que desfavorece oportunidades de prevenção e tratamento, culminando em diagnósticos tardios e apresentações clínicas graves nesse grupo (16,17,18).

Nesta análise, identificou-se que a ausência de informações sobre os dados demográficos e socioeconômicos nas notificações de sífilis é um achado que demonstra como é difícil a caracterização fidedigna dessa doença em pessoas idosas, o que compõe um dos entraves para o planejamento de políticas eficazes nessa população. Trata-se de um fator limitante na avaliação das interações das características socioeconômicas com o risco de sífilis na terceira idade. Em outras populações, como gestantes, sabe-se que a prevalência elevada está associada a fatores de vulnerabilidade socioeconômica, como baixa escolaridade e raça/cor da pele preta ou parda (19). Essa associação entre sífilis e determinantes sociais é um padrão global, em que a prevalência aumenta em populações com menor renda e nível de escolaridade (20,21). Além do risco de infecção, barreiras socioeconômicas, como a dificuldade de acesso a serviços de saúde, comprometem a adesão e a conclusão do tratamento, um problema documentado globalmente e no Brasil (22). 

Nesta análise, as regiões Sul e Centro-Oeste lideraram nas taxas de detecção, o que diverge de estudos anteriores que apontavam maior protagonismo do Sudeste (6,7,18). Essa mudança pode ser explicada, em parte, pela metodologia mais precisa de cálculo demográfico utilizada neste estudo. Por uma perspectiva ampla, essa assimetria regional ocorre com América Latina, sendo uma das regiões com maior prevalência de sífilis entre homens que fazem sexo com homens no mundo, o que reforça a importância de monitorar esses epicentros regionais (10,14). A predominância de casos no sexo masculino também foi evidente, um padrão consistente com a literatura (6,4). A concentração de casos nessas regiões sul-americanas e nos grupos é alarmante, especialmente pela sinergia entre sífilis e HIV, em que a coinfecção acelera a progressão de ambas as doenças e aumenta o risco de transmissão do HIV (9,23,24,25).

A avaliação deste estudo demonstrou a ocorrência de uma mudança de tendência após 2020, quando o crescimento acelerado deu lugar a um platô em níveis elevados na maioria dos grupos. Esse novo comportamento da doença pode ser reflexo de dinâmicas sexuais alteradas pela pandemia de covid-19, mas também pode indicar uma subnotificação crônica que estabilizou os casos de sífilis. A pandemia sobrecarregou os sistemas de saúde globalmente, o que impactou a capacidade de vigilância e de notificação de outras doenças (26). Apesar da estabilização na maioria dos estratos, observaram-se focos de crescimento persistentes em subgrupos específicos, como homens de 70-79 anos no Sul e no Sudeste e pessoas idosas com mais de 80 anos no Norte, o que indica que a vigilância epidemiológica precisa ser intensificada nessas populações de maior vulnerabilidade.

É importante considerar em que medida as taxas de detecção poderiam variar caso fossem adotadas as projeções intercensitárias oficiais do IBGE. Como tais projeções subestimaram a população idosa em vários estados quando comparadas ao censo de 2022, especialmente nas regiões Sul e Centro-Oeste, o uso desses valores como denominador resultaria em taxas de detecção artificialmente mais elevadas. Apesar dessa potencial variação numérica, análises exploratórias mostraram que o padrão geral de crescimento até 2020, seguido de estabilização no período subsequente, permaneceria inalterado. Assim, embora diferentes estratégias de estimativa populacional possam modificar ligeiramente os valores absolutos das taxas, a direção das tendências e as conclusões principais deste estudo demonstram robustez frente às discrepâncias demográficas observadas.

A assimetria de gênero, com taxas de detecção superiores em indivíduos do sexo masculino, é um dos achados centrais deste estudo. Comportamentos de risco e a negligência ao uso de preservativos, muitas vezes motivados por uma percepção equivocada de que não se corre o risco de ser infectado por IST, podem explicar essa disparidade (6,7,27,4). A análise de tendência pós-2020, no entanto, revela que a mudança para um platô de estabilização foi comum a ambos os sexos na maioria das faixas etárias (Tabela Suplementar 1). A principal exceção ocorreu no grupo de 70 a 79 anos, em que a assimetria se acentuou: enquanto as mulheres apresentaram estabilidade, os homens mantiveram uma tendência de crescimento. Essa persistência de crescimento em homens idosos é preocupante, pois eleva o risco de desfechos graves e de coinfecção com o HIV, ampliando o impacto na saúde pública. Embora as taxas de infecção entre as mulheres sejam menores, sua permanência em níveis elevados ao longo do tempo reflete vulnerabilidades específicas, como tabus sobre a sexualidade na terceira idade e uma menor percepção de risco, que dificultam a busca por prevenção e diagnóstico (28,29).

Esta análise utiliza dados secundários do SINAN e está sujeita a limitações como disponibilidade dos dados limitada até o mês de dezembro do ano de 2023, subnotificação e inconsistências (6,7). Adicionalmente, a acurácia diagnóstica é uma variável importante. Algoritmos tradicionais de diagnóstico, que iniciam com testes não treponêmicos, podem ter sensibilidade reduzida para casos de sífilis primária e latente (30). Análises mundiais também mostram que o uso de apenas um tipo de teste (treponêmico ou não treponêmico) tende a superestimar a prevalência em comparação com o padrão-ouro de teste duplo (9), o que adiciona uma camada de complexidade à interpretação de dados de vigilância. 

A sub-representação de idosos, especialmente acima de 80 anos, pode resultar da exclusão desse grupo da testagem. Tal prática baseia-se na percepção de ausência de risco. Essa lacuna limita inferências sobre infecções em idades avançadas. Além disso, os dados utilizados contemplam apenas o período até 31 de dezembro de 2023, correspondente ao último conjunto de notificações consolidadas, auditadas e disponibilizadas publicamente pelo Ministério da Saúde no momento da extração. Informações referentes a 2024 e 2025 estavam indisponíveis ou em fase de qualificação, portanto, não atendiam aos critérios de completude exigidos para análises de série temporal. A ausência de dados de 2024 e 2025 reflete limitações operacionais do sistema de vigilância e não uma restrição metodológica imposta pelos autores. Apesar disso, o estudo contribui para ampliar a compreensão da sífilis acerca das populações idosas. 

A detecção de sífilis em pessoas idosas no Brasil apresentou uma tendência de crescimento até 2020, estabilizando em um platô de taxas elevadas no período de 2020 a 2023. Ocorreram maiores taxas de detecção concentradas nas regiões Sul e Centro-Oeste, predominância no sexo masculino e, principalmente, na faixa etária de 70 a 79 anos. A análise pós-2020 revela a persistência de focos de crescimento em subgrupos específicos, o que indica contínua vulnerabilidade. Tal constatação reforça a necessidade urgente de repensar políticas de saúde sexual para pessoas idosas, além de estratégias de prevenção e rastreamento direcionadas para controlar a disseminação da sífilis. 

No contexto do Sistema Único de Saúde (SUS), sistema público de saúde brasileiro, a tendência de crescimento pode indicar risco semelhante para outras infecções sexualmente transmissíveis, como HIV, o que torna essencial o fortalecimento da vigilância integrada. Para lidar com esse cenário, é indispensável investir na qualificação de profissionais da atenção primária à saúde e desenvolver campanhas educativas específicas relacionadas à sexualidade na terceira idade, além de expandir o acesso a testes rápidos e tratamento oportuno em unidades básicas de saúde.

Portanto, seria válida criar linhas de cuidado em saúde sexual para pessoas idosas, integradas às redes de atenção à saúde e a incorporação de testagem de IST sempre que adequado. Como agenda futura, pesquisas devem explorar os determinantes sociais e comportamentais associados à aceleração das taxas no período pós-pandêmico e avaliar a efetividade de intervenções específicas no âmbito do SUS. Além disso, deve-se ampliar os horizontes no âmbito da pesquisa para avaliar com maior clareza o impacto da sífilis na saúde das pessoas idosas pela perspectiva clínica dentro do SUS, o que demandaria ampliar a coleta de dados para avaliar de modo mais significativo o percentual de cura e a eficácia do tratamento.

## Data Availability

O conjunto de dados de replicação que apoia os achados deste estudo está publicamente disponível no repositório SciELO Data sob o identificador de objeto digital (DOI): https://doi.org/10.48331/SCIELODATA.NQ5H7D. Os dados epidemiológicos de sífilis também podem ser acessados pelo portal do Departamento de Informática do Sistema Único de Saúde, na seção do Sistema de Informação de Agravos de Notificação, no seguinte endereço: http://www.datasus.gov.br. Os dados demográficos também podem ser consultados no portal do Instituto Brasileiro de Geografia e Estatística (IBGE) em: https://www.ibge.gov.br.
